# Getting Started in Structural Phylogenomics

**DOI:** 10.1371/journal.pcbi.1000621

**Published:** 2010-01-29

**Authors:** Kimmen Sjölander

**Affiliations:** Department of Bioengineering, University of California, Berkeley, Berkeley, California, United States of America; Princeton University, United States of America

*Structural phylogenomics* refers to the combined use of evolutionary and structural information in a bioinformatics analysis. The term *phylogenomics* refers to two distinct tasks: reconstructing a species phylogeny using multiple genes (for a review, see [1]) and predicting protein function by estimating the evolutionary history of a family of related sequences (i.e., a gene tree or multi-gene tree including gene duplication events) [2]–[4]. In this “Getting Started” article, we focus on the latter task, restricting our discussion to the construction and analysis of phylogenetic trees for amino acid data, and including protein structure data and structure prediction to improve the accuracy of functional annotation. We address the following questions: Why perform a complicated structural phylogenomic analysis when simpler approaches are available? What are the fundamental underlying assumptions of this approach, and what are the implications of any conflicts with these assumptions? What technical challenges do we need to address to achieve the full potential of these ideas?

Structural phylogenomics is essentially a philosophy rather than a particular methodology. The intimate link between protein structure and function is well known; structural phylogenomics brings evolutionary modeling into this mix, to elucidate and exploit the connection between evolutionary events and innovations in protein function and structure.

Protein superfamilies evolve novel functions and structures through mutations at key positions, gene duplication, internal repeats, and gene fusion and fission events. Many proteins are composed of multiple structural *domains*—independently folding globular building blocks that can be found in different domain architectures (or *domain organizations*)—allowing a kind of mix-and-match grab-bag of function and structure. The result of these evolutionary innovations is a multiplicity of biological functions and structures that contribute to the diversity of life forms. Consider G protein–coupled receptors (seven-transmembrane receptors found in many eukaryotic species); over 800 have been found in the human genome alone. These genes have diverged from their common ancestor through repeated duplication events, allowing them to recognize hundreds of different ligands and to participate in distinct biological processes. Tracking the evolution of a protein superfamily within and across different species and overlaying the phylogenetic tree with experimental data can allow highly nuanced predictions of function and structure for uncharacterized proteins.

A phylogenomic analysis, as originally defined [2], is designed to address the systematic errors associated with the standard protocol in functional annotation of proteins: annotation transfer from the top hit in a database search [5]–[8]. A typical phylogenomic analysis involves (i) selecting a dataset (clustering homologs), (ii) constructing a multiple sequence alignment, (iii) estimating a phylogenetic tree, (iv) analyzing the tree to distinguish between orthologs (sequences related by speciation, and thus presumed to share a common function) and paralogs (sequences related by gene duplication from a common ancestor and thus potentially divergent in function), (v) overlaying the tree with experimental data and biological annotations from resources such as the manually curated SwissProt database, and finally, (vi) inferring the function(s) of individual sequences in the family based on their placement in the tree.

Structural information can be useful at various points in a phylogenomic inference of function. For instance, most annotation transfer protocols do not differentiate between homologs having only local similarity and those aligning along their entire lengths, and can thus result in errors in annotation. Incorporating domain architecture information (e.g., through the use of PFAM analysis), or using a structural phylogenomic clustering method such as FlowerPower [9], can reduce these errors. Structural information can also be used to identify individual domains in multi-domain proteins for separate phylogenetic analyses. Thus, it is possible to reconstruct the evolutionary history of a kinase domain; kinase domains can be extracted from proteins containing these domains for phylogenetic reconstruction (e.g., [10]). These *semi-global* (sometimes called “glocal”) clustering techniques and alignments are also used to construct hidden Markov models (e.g., as in the popular PFAM resource) for use in identifying important functional or structural domains in novel sequences. While internal nodes (evolutionary branch points) of protein superfamily phylogenies typically represent speciation and duplication events, in cases where a structural domain-based phylogeny includes sequences with different domain architecture, internal nodes of the tree may also represent gene fusion and fission events; this complicates a phylogenetic analysis, but can yield very powerful insights into the functional roles of individual domains.

A fundamental tenet of phylogenomic inference of function is that the evolutionary tree is correct. Is this assumption reasonable? In our experience estimating phylogenetic trees for protein superfamilies in the PhyloFacts Phylogenomic Encyclopedias [11], we have observed disagreements in topology produced by different phylogenetic tree estimation methods for the same datasets, particularly at branches joining paralogous groups. Clearly, not all can be correct. Which methods are most appropriate for protein superfamily phylogeny estimation? Given that protein superfamilies can contain many hundreds or thousands of members, are fast methods (such as neighbor-joining) sufficiently accurate, or do we need to use computationally expensive methods such as MrBayes, maximum likelihood, or maximum parsimony? Differences between methods can result in very different functional annotations using phylogenomics when no orthologs with experimentally supported function can be identified for sequences of interest; in these cases it may be necessary to transfer annotations from subtree neighbors (sequences that are siblings in the tree but not strictly orthologous) [12].

Many factors can affect phylogenetic tree topology accuracy. First, dataset selection (how homologs are selected) can affect phylogenetic accuracy. It is not uncommon to see phylogenetic trees restricted to sequences from whole (fully sequenced) genomes, or from a subset of available species. These restrictions in dataset selection may be convenient, but can cause problems in the estimated phylogeny due to sparse taxon sampling [13]. Second, phylogenetic reconstruction methods generally assume that the input multiple sequence alignment is correct. That is, for each column in the alignment, every character in that column descends from an ancestral character (termed *positional homology*). However, numerous studies have shown that sequence alignment accuracy drops sharply with evolutionary divergence [14]. Manual editing of an alignment and the incorporation of structural information can help reduce these errors, but may not resolve all potential problems in an alignment.

To mitigate the impact of errors in a multiple sequence alignment, alignment masking is typically performed to remove columns of uncertain homology prior to phylogenetic tree estimation. However, alignment masking protocols appropriate for species phylogeny estimation, where the input is a concatenated alignment of many genes/proteins (so-called *genome trees* or gene matrix approaches [1]), may be inappropriate for protein superfamily phylogeny estimation. While the former may have thousands of columns and a relatively small fraction will be masked due to low overall sequence divergence, the latter may have at most a few hundred columns and a relatively large fraction of columns are likely to be masked. Such stringent masking protocols can reduce the effective information available for a phylogenetic reconstruction. In addition, positions targeted for masking due to high divergence may, in fact, be essential for tree topology accuracy: positions that vary across the family as a whole but are conserved within closely related clades may be required to get the phylogenetic groupings correct. The SATCHMO (simultaneous alignment and tree construction using hidden Markov models) method addresses this issue using agglomerative clustering and profile–profile alignment to estimate a tree topology and multiple sequence alignment simultaneously, and performs alignment masking within each subtree separately to mask positions appearing to have structurally diverged across the sequences that descend from a node [15].

A third challenge is that the extreme sequence, structural, and functional divergence observed in most protein superfamilies may not be handled effectively by phylogenetic tree reconstruction methods. Even when a phylogenetic method allows for shifts in lineage- and site-specific rate variation, extreme rate variation—especially when coupled with probable alignment errors across highly divergent groups—may make it difficult to determine the correct branching order between distantly related clades. For these reasons, errors in tree topology must be expected at the coarse branching order of a protein superfamily phylogeny.

All these issues contribute to regions of a protein family phylogeny that may be poorly resolved (e.g., have low bootstrap support), or in which different phylogenetic methods may disagree on the branching order. Given these possible problems, how do we evaluate phylogenetic tree methods for use in analysis of protein superfamilies?

Phylogenetic tree estimation methods have traditionally been evaluated in two ways: using simulation studies and comparing inferred trees and trusted phylogenies based on fossil data and morphological characteristics. Phylogenetic simulation protocols do not currently model gene duplication events and structural and functional changes, but could presumably be modified to do so. However, while we do not (generally) have the equivalent of fossil evidence for gene families, we do have the rough equivalent of morphological data: abundant 3D protein structures; assays for biochemical function; experimental data indicative of biological process and pathway association, cellular localization, protein–protein interaction, and so on. We propose that these data could be used to evaluate protein superfamily phylogenies estimated from sequence information. Our fundamental assumption in proposing the use of these experimental data to evaluate protein superfamily phylogenies is the following: since evolution is primarily conservative of function and structure, a phylogenetic tree that clusters functionally and structurally similar proteins ought to be more accurate (that is, correspond more closely to the true evolutionary history) than one that does not. Unfortunately, this approach has two fundamental limitations. First, functional similarity is not easily quantifiable. Second, evaluating phylogenies based on agreement with annotated function is problematic because of the prevalence of annotation errors and the paucity of experimentally supported annotations.

Fortunately, protein structure has several attributes that make it an appealing basis for evaluation of phylogenetic trees. First, structural similarity correlates closely with evolutionary distance, with closely related proteins having higher structural similarity than more distantly related proteins [14]. Second, structural similarity is easily quantified, and numerous software tools exist to superpose protein 3D structures and compute various scores based on that superposition. Third, many protein families have representative 3D structures for different subtypes. This suggests that phylogenetic methods could be compared on the basis of their ability to cluster structurally similar proteins on the tree (i.e., structural similarity ought to correspond roughly to proximity in the tree).

In this article, we are clearly advocating a pragmatic approach to evaluating the effectiveness and utility of phylogenetic tree methods, rather than one that is based on some theoretical or ideological agenda. Simply put, we need a concrete measure of the utility of phylogenetic methods for functional inference. Whether these estimated phylogenies correspond to the *true* tree may not be known (or even knowable), but we can assess the predictive power of phylogenetic methods for their actual use in practice.

If phylogenetic methods have limitations with respect to protein superfamily analysis, how can we improve them? In particular, is it appropriate to use protein structure or other experimental data to improve phylogenetic reconstruction accuracy? For instance, it has been shown that inclusion of phylogenetic information improves the specificity and sensitivity of bioinformatics methods for numerous tasks, including enzyme active site identification [16],[17], prediction of ligand-binding residues, protein structure prediction [18], functional subfamily identification, and remote homolog detection [19]. If using phylogenetic information improves the prediction of protein structure and function, it must be because there is a relationship between evolutionary processes and functional and structural divergence. If so, shouldn't the improvement also work in the reverse direction? That is, shouldn't we expect an improvement in phylogenetic tree topology accuracy through the inclusion of experimental data relevant to protein structure and function?

In our experience, orthologous groups are typically clustered correctly by most phylogenetic estimation methods. Where methods disagree is at the coarse branching order between paralogous genes. (These regions of phylogenetic trees tend, not surprisingly, to also have low bootstrap support.) At these evolutionary branch points, sequence information may be insufficient for phylogenetic resolution, and the use of structural data might prove helpful. If solved structures are available for some of these subtrees, we could use these data to bias the tree topology estimation (i.e., to favor joining subtrees whose structures are more superposable). This joint analysis of sequence and structural characters is enabled by MrBayes (as shown in [10]). Of course, the utility of any such approach could not be evaluated on the basis of agreement with structural superposition data, because of circular reasoning.

While structural phylogenomics is just one of many methods for predicting protein function (see [20] for a review of automatic function prediction methods), and has distinct technical challenges, we propose that even a minimal phylogenomic analysis is better than none. As Sir Winston Churchill said in a speech to the House of Commons, 11 November 1947: “Democracy is the worst form of government, except for all those other forms that have been tried from time to time.” Even if structural phylogenomics may not be a *complete* solution to the problem of protein function prediction, it has been shown to provide significantly higher precision in functional annotation than many other approaches to this task, and provides a unique framework for investigating the changes in protein function and structure explored by evolution.
